# Targeting immune checkpoints in anti-neutrophil cytoplasmic antibodies associated vasculitis: the potential therapeutic targets in the future

**DOI:** 10.3389/fimmu.2023.1156212

**Published:** 2023-04-06

**Authors:** Menglu Pan, Huanhuan Zhao, Ruimin Jin, Patrick S. C. Leung, Zongwen Shuai

**Affiliations:** ^1^ Department of Rheumatology and Immunology, First Affiliated Hospital of Anhui Medical University, Hefei, China; ^2^ Division of Rheumatology/Allergy and Clinical Immunology, University of California, Davis, Davis, CA, United States; ^3^ Inflammation and Immune Mediated Diseases Laboratory of Anhui Province, Hefei, China

**Keywords:** antineutrophil cytoplasmic autoantibody associated vasculitis, immune checkpoint, co-stimulatory signal pathway, co-inhibitory signal pathway, T cells, immunotherapy

## Abstract

Anti-neutrophil cytoplasmic autoantibodies (ANCA) associated vasculitis (AAV) is a necrotizing vasculitis mainly involving small blood vessels. It is demonstrated that T cells are important in the pathogenesis of AAV, including regulatory T cells (Treg) and helper T cells (Th), especially Th2, Th17, and follicular Th cells (Tfh). In addition, the exhaustion of T cells predicted the favorable prognosis of AAV. The immune checkpoints (ICs) consist of a group of co-stimulatory and co-inhibitory molecules expressed on the surface of T cells, which maintains a balance between the activation and exhaustion of T cells. CD28, inducible T-cell co-stimulator (ICOS), OX40, CD40L, glucocorticoid induced tumor necrosis factor receptor (GITR), and CD137 are the common co-stimulatory molecules, while the programmed cell death 1 (PD-1), cytotoxic T lymphocyte-associated molecule 4 (CTLA-4), T cell immunoglobulin (Ig) and mucin domain-containing protein 3 (TIM-3), B and T lymphocyte attenuator (BTLA), V-domain Ig suppressor of T cell activation (VISTA), T‐cell Ig and ITIM domain (TIGIT), CD200, and lymphocyte activation gene 3 (LAG-3) belong to co-inhibitory molecules. If this balance was disrupted and the activation of T cells was increased, autoimmune diseases (AIDs) might be induced. Even in the treatment of malignant tumors, activation of T cells by immune checkpoint inhibitors (ICIs) may result in AIDs known as rheumatic immune-related adverse events (Rh-irAEs), suggesting the importance of ICs in AIDs. In this review, we summarized the features of AAV induced by immunotherapy using ICIs in patients with malignant tumors, and then reviewed the biological characteristics of different ICs. Our aim was to explore potential targets in ICs for future treatment of AAV.

## 1 Introduction

Anti-neutrophil cytoplasmic antibody (ANCA) associated vasculitis (AAV) is a group of life-threatening diseases, characterized by necrotizing inflammation of small blood vessels, with pauci-immune complex depositions. According to different clinical manifestations, it was mainly divided into three types, including granulomatosis with polyangiitis (GPA), microscopic polyangiitis (MPA), and eosinophilic granulomatosis with polyangiitis (EGPA) (1–4). ANCA is composed of series of autoantibodies identifying their autoantigens in neutrophil plasma, including proteinase-3 (PR3) and myeloperoxidase (MPO) which may be expressed on activated neutrophils. GPA, mainly associated with PR3-ANCA, usually affects the sinuses, the lung, and the kidney with specific granulomatous inflammation. In contrast to GPA, MPA frequently damages the lung and the kidney with necrotizing vasculitis. In general, MPO-ANCA is predominantly detected in patients with MPA and EGPA. The clinical characteristics of EGPA include asthma, eosinophilia, and vasculitis (5, 6), but it is much less common than GPA and MPA. The distribution of AAV might be influenced by geographical and race. In east Asia, especially in China and Japan, MPA with MPO-ANCA is the predominant AAV, whereas in Europe, such as the UK and France, GPA with PR3-ANCA is the more common AAV (7, 8).

**Figure 1:**
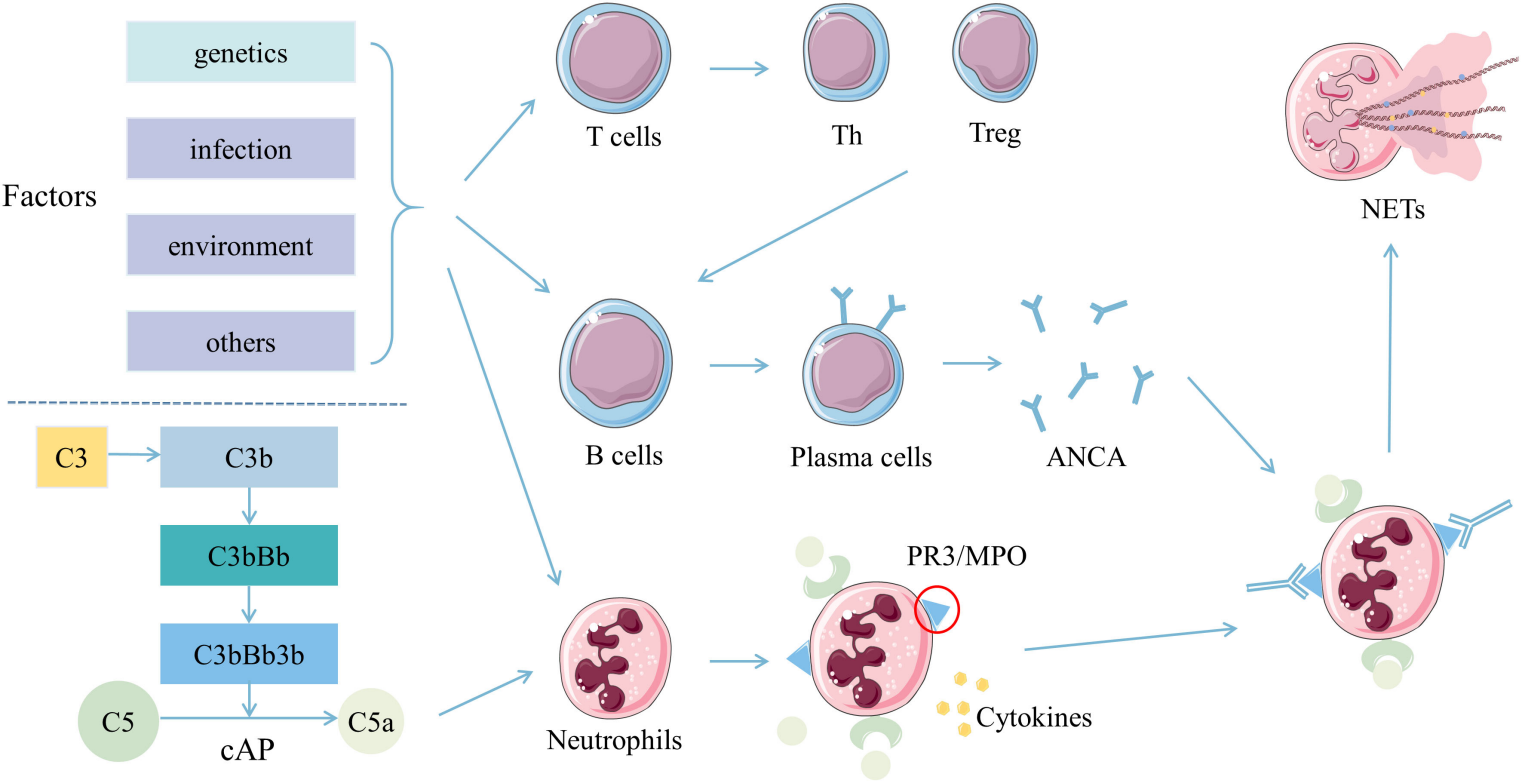
Pathogenesis of ANCA associated vasculitis. T cells, B cells, and neutrophils were activated by multiple stimulations in the background of genetic susceptibility. Th and Treg cells differentiated from T cells synergistically promote B cells to develop into plasma cells, and produce ANCA. ANCA then combined with PR3 or MPO expressed on neutrophils pre-activated by inflammatory cytokines. Also, C5a derived from activated cAP might combine with the C5a receptor on neutrophils. Neutrophils activated by ANCA, C5a and various cytokines might produce more NETs conducive for the inflammatory response and adaptive immunity, ultimately leading to clinical damages. Abbreviations: ANCA, anti-neutrophil cytoplasmic autoantibodies; C5a, fragment a of the fifth complement; cAP, complement alternative pathway; MPO, myeloperoxidase; NETs, neutrophil extracellular traps; PR3, proteinase-3; Th, helper T; Treg, regulatory T.

Although the exact etiopathogenesis of AAV remains unclear, studies have demonstrated that some factors, such as T and B cells, ANCA, the complement alternative pathway (cAP), and neutrophil extracellular traps (NETs), might play various important roles in the pathogenesis (**Figure 1**). On the genetic background, the interaction of infections, environmental and other factors might activate T cells, which could help B cells develop into plasma cells. Meanwhile, neutrophils could also be activated and express PR3 and MPO to combine with the ANCA secreted by plasma cells (9–11). The fragment a of fifth complement (C5a) produced by activating cAP could connect with the C5a receptor on neutrophils (12, 13). The combinations of these factors on neutrophils could activate the neutrophils further to promote their degranulation and NETs production, intensify respiratory burst, injury the vascular endothelium, accelerate the inflammatory response, and ultimately lead to clinical damages of AAV (11, 13–15).

T cells play essential and pivotal roles in autoimmunity. Studies have revealed that subgroups of T cells, including regulatory T cells (Treg) and helper T cells (Th), especially follicular Th cells (Tfh), Th2 and Th17, were involved in the pathogenesis of AAV (16–19). The exhaustion of T cells could predict the favorable prognosis of AAV (20). Notably, the activation of T cells requires two signals. The first signal is traditional T cell receptor (TCR) signaling triggered by the recognition between TCR and specific peptides from the major histocompatibility complex (MHC) on the surface of antigen-presenting cells (APC). Immune checkpoint (IC) molecules transmit the second signal. ICs are a class of surface proteins to provide co-stimulatory or co-inhibitory signals by combining with the corresponding receptors or ligands on the surface of APCs (21, 22). Currently, immune checkpoint inhibitors (ICIs) have been used in treating various malignant tumors (23–26). However, 3.5% of patients treated with ICIs occurred rheumatic disease (27), suggesting that targeting ICs might have potential values in the treatment of rheumatic diseases. In this review, we summarized the association between ICIs and AAV, focused on the characteristics of ICs, and explored the potential therapeutic prospect of targeting ICs in AAV.

## 2 The association between ICIs and AAV

Currently, there are three types of ICIs in blocking co-inhibitory pathways, targeting programmed cell death 1 (PD-1) (nivolumab, pembrolizumab and cemiplimab), programmed death ligand 1 (PD-L1) (avelumab, atezolizumab and durvalumab), and cytotoxic T lymphocyte-associated molecule 4 (CTLA-4) (ipilimumab and tremelimumab). As the signal transductions of co-inhibitory were blocked, T cells could be activated, and then the tumor immunity could be enhanced (28, 29). Consequently, the inflammatory response might also be increased due to the activation of T cells, resulting in most patients developing immune-related adverse events (irAEs). The damages of irAEs could involve multiple organs, including but not limited to the skin, the gastrointestinal tract, the lung, and the kidney (29, 30). It is shown that CTLA-4 inhibitors may induce more irAEs than PD-1 inhibitors, and the combination of these two kinds of inhibitors can further increase the incidence of irAEs (31, 32).

Despite the rheumatic irAEs (Rh-irAEs) are not common in all irAEs, rheumatologists are still concerned about these Rh-irAEs. It was reported that the frequent Rh-irAEs were inflammatory arthritis and inflammatory myopathy. ICIs-induced vasculitis is less common than other rheumatic diseases and mainly affects the medium and large arteries (27, 29, 33). In **Table 1**, we summarized the cases of ICIs-induced AAV reported to date (23–25, 34–40). Interestingly, even with ICIs treatment, some AAV patients in remission did not relapse (41–43). Therefore, we speculated that the ICs molecules might be involved in the pathogenesis of AAV. What is more, different ICs might dominate in various stages.

**Table 1:** Demographic characteristics and clinical data of patients with ICIs-induced AAV.

Case	Age/Gender	ICIs	Target	irAEs	Clinical Features	Treatments	Outcome
Kato, et al. (34)	N	nivolumab	PD-1	AAV	–	–	–
Hung, et al. (35)	66/female	ipilimumab and nivolumab	CTLA-4 and PD-1	GPA	headache, polyarthralgia, proteinuria, and hemoptysis	glucocorticoid, rituximab and one dose of infliximab	chronic kidney disease
Uner, et al. (36)	65/male	pembrolizumab	PD-1	AAV	diarrhea, increased creatinine, microscopic hematuria, and proteinuria	glucocorticoid and rituximab	chronic kidney disease
Harada, et al. (37)	65/male	nivolumab	PD-1	EGPA	asthma, eosinophilia, dyspnea on exertion, arthritis.	glucocorticoid	remission
Mamlouk, et al. (38)	70/male	tremelimumab	CTLA-4	MPA	microscopic hematuria, pyuria, and proteinuria	glucocorticoid, plasmapheresis, and rituximab	chronic kidney disease
Roger, et al. (23)	34/female	nivolumab	PD-1	EGPA	asthma, eosinophilia, arthritis, and pansinusitis	glucocorticoid	–
Nabel, et al. (25)	56/male	pembrolizumab	PD-1	GPA	arthritis, cough, emesis, and diffuse expiratory wheezes in the right lung	glucocorticoid and rituximab	remission
Sibille, et al. (39)	64/male	pembrolizumab	PD-1	GPA	myositis, dyspnea, and arthritis	glucocorticoid	remission
Heo, et al. (24)	56/male	pembrolizumab	PD-1	GPA	rash, fever, arthralgia, myalgia, increased creatinine, microscopic hematuria, and proteinuria	glucocorticoid and hemodialysis	remission
van den Brom, et al. (40)	56/female	ipilimumab and pembrolizumab	CTLA-4	GPA	fever, arthritis, cutaneous vasculitis, and pulmonary nodules	glucocorticoid and cyclophosphamide	remission

AAV, anti-neutrophil cytoplasmic autoantibodies associated vasculitis; CTLA-4, cytotoxic T lymphocyte-associated molecule 4; EGPA, eosinophilic granulomatosis with polyangiitis; GPA, granulomatosis with polyangiitis; ICIs, immune checkpoint inhibitors; MPA, microscopic polyangiitis; PD-1, programmed cell death 1.

## 3 Co- stimulatory signal pathways

**Figure 2:**
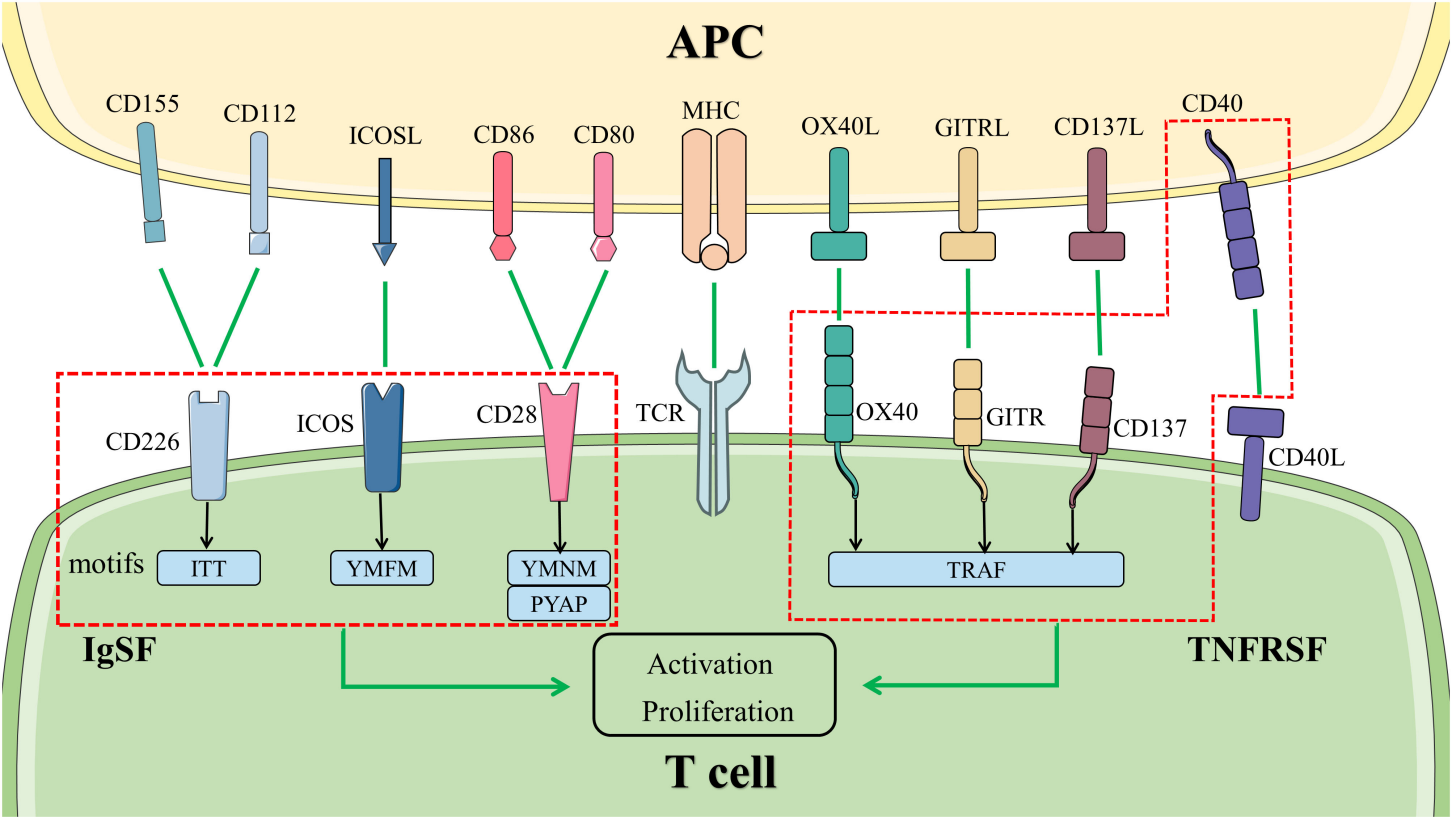
Co-stimulatory signal pathways in T cells. The activation and proliferation of T cells at least need two signals. The first signal is provided by the binding of TCR to MHC with antigenic polypeptide processed by APC. Co-stimulatory molecules bind to the ligand or receptor presented by APCs, transmitting the second signal. The molecules in the left box are members of IgSF, activating the signal pathways downstream by different motifs in the cytoplasmic tail. The members of TNFRSF are represented in the right box. They bind to TRAF to activate the signal pathways downstream. Abbreviations: APC, antigen-presenting cell; GITR, glucocorticoid induced tumor necrosis factor receptor; ICOS, inducible T-cell co-stimulator; IgSF, immunoglobulin superfamily; MHC, major histocompatibility complex; TCR, T cell receptor; TNFRSF, tumor necrosis factor receptor superfamily; TRAF, tumor necrosis factor receptor-associated factor.

The co-stimulatory molecules expressed on the surface of T cells contained CD28, inducible T-cell co-stimulator (ICOS), OX40, and others (**Figure 2**). They will be discussed in detail below.

### 3.1 CD28 signal pathway

CD28 is a member of the immunoglobulin (Ig) superfamily (IgSF) with typical Ig variable (IgV) domains. This transmembrane protein of 44 kDa is composed of a disulfide-linked homodimer. It was involved in the formation of immunologic synapses, the phosphorylation of proteins, and the remodeling of actin in T cells. Consequently, T cells were activated and produced cytokines (44–46). CD28 signal transduction is relied on two motifs in its cytoplasmic tail: YMNM and PYAP. The phosphorylated Src homology-2 (SH2) domain in the proximal YMNM motif binds the p85 subunit of phosphatidylinositol 3-kinase (PI3K), the growth factor receptor-bound protein 2 (GRB2), and the GRB2-related adapter protein 2. The distal PYAP motif combines with the lymphocyte cell-specific protein-tyrosine kinase (LCK) and GRB2. Their bindings could activate the downstream targets, including nuclear factor-κB (NF-κB), nuclear factor of activated T cells (NFAT), mammalian target of rapamycin (mTOR), and mitogen-activated protein kinase to affect cell cycle progression, apoptosis, and especially interleukin (IL)-2 transcription (47).

CD80 and CD86 are cognate ligands of CD28, which are mainly expressed on the surface of APCs, such as B cells, dendritic cells (DCs), and monocytes (17, 22, 48). However, there are some differences between their features and functions. CD80, which is existed as a dimer, is expressed rarely on resting B cells whereas the expression of CD86, which is in the form of a monomer, is higher than CD80. When B cells were activated, the density of CD86 on its surface increased early and even more compared with CD80 (49, 50). In animal experiments, it is demonstrated that in CD86-deficient mice, neither the antibody isotypes were switched nor germinal centers (GCs) were formed, whereas it is contrary in CD80-deficient mice (51). Furthermore, because CTLA-4, a co-stimulatory molecule with highly homologous to CD28, has a higher affinity for CD80 and CD86, it competitively inhibits the bindings of CD28 to CD80 and CD86, and actually restrains the cellular immune responses (52).

CD28 signal pathway plays a vital role in vasculitis. Zhang et al. constructed human artery-severe combined immunodeficiency mice chimeras with peripheral blood mononuclear cells from patients with giant cell arteritis (GCA) to induce vasculitis. Blocking the CD28 signal pathway significantly disrupted T-cell metabolic fitness and inhibited the remodeling of the vessel wall (53). In Takayasu’s arteritis (TAK), another large vasculitis, the active patients had higher mRNA levels of CD28 than the inactive patients (54). In patients with active GPA, T cells had a higher proliferative response to the stimulation of CD2/CD28 than healthy controls (HCs) (55). In addition, the expressions of CD80 and CD86 were also significantly increased in CD19^+^ B cells from patients with frequently relapsing EGPA (17). Besides ICs in cell membranes, soluble ICs have received attention gradually. Soluble ICs were produced by the proteolytic cleavage of extracellular regions, or by alternative splicing (56). Elevated soluble CD28(sCD28) levels was observed in sera samples in patients with active AAV. Such increase in was significantly positively correlated with disease activity markers, such as the Birmingham Vasculitis Activity Score, C-reactive protein, and erythrocyte sedimentation rate (57). Noteworthy, sCD28 level decreased when AAV patients from the active state became inactive after treatment (57), suggesting that sCD28 might play a potential immunopathological role in AAV and could be a novel biomarker to evaluate disease activity. Therefore, targeting the CD28 signal pathway may be effective for AAV. Abatacept, a CTLA-4-Ig fusion protein composed of the ligand-binding domain of CTLA-4 and the modified Fc portion of IgG, can block CD28 signal transduction by bindings to CD80 and CD86 (58). An open-label trial reported that Abatacept had improved the disease condition in patients with non-severe relapsing GPA (59). We prefer to inhibit the CD28 signal transduction directly. FR104 is a pegylated antigen-binding fragment (Fab) antibody. In the non-human primate (NHP) graft-versus-host disease (GVHD) model, the survival of GVHD-free was improved by FR104/sirolimus. Still, the overall survival was not improved because of the sepsis and a paralyzed interferon (IFN)-γ response in some patients without GVHD (60). In conclusion, it has clinical significance for targeting the CD28 signal pathway. Nevertheless, the challenge is to develop a more effective anti-CD28 antibody with fewer side effects.

### 3.2 ICOS signal pathway

Even though ICOS and CD28 belong to the same family, they still have several differences. Firstly, ICOS is expressed on the surface of activated T cells. The activation of T cells by TCR and CD28 signal is essential for the expression of ICOS. Then, ICOS can promote the activation of T cells further (61). Secondly, there is only one particular YMFM motif in the cytoplasmic tail of ICOS. YMFM binds to the p50α and p85 subunits of PI3K and tends to recruit the former. Subsequently, the AKT signal enhances markedly. The ICOS-PI3K-AKT pathway promotes the expression of cytokines as well as induces the formation of Tfh cells. Tfh cells migrate into the follicles, maintain in GCs, and promote the differentiation of B cells into plasma cells to secrete ANCA (21, 62–64). Thirdly, ICOS could combine with the ICOS ligand (ICOSL) instead of CD80 and CD86 (65).

ICOSL is expressed on the surface of B cells, macrophages, fibroblasts, muscle cells, podocytes, and other cells (65–68). Several factors regulated the expression of ICOSL on the surface of B cells. B cell receptor (BCR) signal reduced the expression of ICOSL on naïve B cells, which affected the formation of Tfh cells. The BCR signal was more substantial, and the reduction of ICOSL was more obvious. This inhibitory response could be reversed by the CD40 signal (69, 70). Similarly, in both NF-κB–inducing kinase (NIK) KO mice and B-cell activating factor belonging to tumor necrosis factor (TNF) receptor (TNFR) family receptor-deficient mice, Hu et al. revealed that the expression of ICOSL decreased significantly. They discovered the recombinant ICOSL-Fc fusion protein could increase the levels of Tfh cells in NIK KO mice, suggesting ICOSL is a target of the noncanonical NF-κB pathway (71).

Several studies focused on the differences of ICOS^+^ Tfh cells in vasculitis (19, 72, 73). Circulating CD4^+^ CXCR5^+^ ICOS^+^ Tfh cells were elevated and correlated with disease activity in patients with Henoch-Schönlein purpura (HSP) (72). The same results were observed in patients with active MPO-AAV (19) and Behcet’s disease (73). In patients with active AAV, the serum concentration of soluble ICOS was also higher than HCs (57). In addition, the production of pro-inflammatory factors was decreased by blocking the ICOS signal (73). AMG 557 is a fully human IgG2 monoclonal antibody (mAb) with a higher affinity to ICOSL that prevents the binding of ICOS and ICOSL. The safety and tolerability of AMG 557 are acceptable in patients with mild, stable systemic lupus erythematosus (SLE) (74). In another phase Ib, randomized, double-blind, placebo-controlled study, the potential efficacy of AMG 557 was evaluated (75). Fewer patients receiving AMG 577 (3 of 10 patients) or placebo (1 of 10 patients) achieved the primary efficacy endpoints. On day 169, compared with the placebo group, more patients in the AMG 557 group showed a ≥4-point improvement in the SLE disease activity index (SLEDAI) (70.0% *vs*. 20.0%, *p*=0.07), indicating the potential efficacy of AMG 557 (75). As mentioned above, ANCA is thought to be the pathogenic antibody of AAV, and the secretion of ANCA by plasma cells is regulated by Tfh cells. Therefore, the investigations on mAb targeting ICOS molecules on the surface of Tfh cells will be one of research directions of AAV treatment in the future.

### 3.3 OX40 signal pathway

The costimulatory molecule OX40 belongs to the TNFR superfamily (TNFRSF), also known as TNFRSF4 or CD134. Similar to other members of TNFRSF, OX40 is a type I transmembrane glycoprotein with four cysteine-rich domains in the extracellular region (76). Different from ICOS, OX40 expressed on CD4^+^ T cells was driven by the TCR signal. After the activation of T cells, the expression of OX40 is promoted by CD28 and CD40 signals (77). In the intracellular region, OX40 binds to TNF receptor-associated factor (TRAF) 2, TRAF3, TRAF 5, and TRAF6, which activates the NF-κB, PI3K-AKT, and NFAT signal pathways downstream. It promotes the survival of T cells and the secretion of cytokines (21, 78).

The only ligand of OX40 is OX40L (also known as TNFSF4, CD252), which is expressed on the surface of DCs, B cells, T cells, vascular endothelial cells (VECs), mast cells, Langerhans cells, and other types of cells (79–84). There is a conserved extracellular TNF homology domain on the OX40L for trimerization (76). OX40L trimer combines with three OX40 molecules to polarize T cells to Th cells, expand Treg cells, sustain the function of memory T cells, and promote the adhesion of activated T cells to VECs (77, 85).

The OX40 signal pathway is vital in rheumatic diseases. In patients with SLE or TAK, the expression of OX40L was enhanced on VECs (82, 86). Besides the expression of OX40, the soluble OX40L was increased in patients with HSP, and both of them were associated with disease activity (87). It was observed in patients with AAV that the expression of CD134 as well as the CD134^+^ T cells were increased. The majority of CD134^+^ T cells mainly secreted TNF-α (88, 89). The results were consistent with *in vitro* studies. So far, several mAbs against OX40 (KHK 4083 and GBR 830) and OX40L (KY 1005) have been developed. KHK 4083 demonstrated the safety and tolerability in patients with mild to moderate plaque psoriasis (90) and moderate to severe ulcerative colitis (UC) (91). In the phase II a study, GBR 830 showed the therapeutic potential for patients with moderate to severe atopic dermatitis (AD) (92). The pharmacological activity of KY 1005 in humans was evaluated. It is considered that targeting OX40L might be effective (93). These findings indicated that the OX40 signal pathway might be a potential therapeutic target in patients with AAV. The problem is that OX40 blockade might inhibit the function of Treg cells, which then leads to disease relapse. In the NHP GVAD model, Tkachev et al. reported that the combined administration with KY 1005 and sirolimus could control the activation of effector T cells while maintaining the reconstitution of Treg cells (94). It seems that combination treatments may be more promising.

### 3.4 Other co-stimulatory signal pathways

There are some other co-stimulatory molecules involving in the activation of T cells as well. We focused on the possibility of targeting CD40L, glucocorticoid induced TNF receptor (GITR), and CD137 for therapy in AAV.

CD40L (also known as TNFSF5 or CD154), mainly expressed in activated T cells and platelets, is the ligand of CD40 (95, 96). CD40, expressed in B cells, monocytes, DCs, and VECs, has similar structures to the other members of TNFRSF (96–99). The connection of CD40 and CD40L regulated Th cells differentiation, maintained GCs response, activated the CD8^+^ cytotoxic T lymphocytes (CTL), and sustained memory CTLs (100). Although the gene polymorphisms of CD40 were not related to the susceptibility of AAV (101), the levels of CD40L and soluble CD40L were raised in AAV patients, which was correlated with disease activity (102). It was a pity that the anti-CD40 mAb BI 655064 and the polyethylene glycol conjugated anti-CD40L Fab’ fragment dapirolizumab pegol (DZP) both did not achieve the expected clinical efficacy in the phase II studies (103, 104). Furthermore, blocking CD40L could lead to severe thromboembolic events. Because of the myocardial infarction and thromboembolic events occurring in patients, the study of BG9588 was terminated (105). In comparison, the CD40L binding protein VIB4920, which lacks an Fc domain, may have more therapeutic potential in AAV. By blocking the downstream CD40 signal, VIB4920 could inhibit the differentiation of plasma cells without platelet aggregation. The safety and efficacy of VIB4920 have been preliminarily demonstrated in patients with rheumatoid arthritis (RA) (106), and further exploration of the clinical efficacy is needed.

GITR (also known as TNFRSF18) and CD137 (also known as 4-1BB or TNFRSF9) are both members of TNFRSF (85). They are expressed in different types of activated T cells, that is, GITR is mainly expressed in Treg cells (107) while CD137 is primarily expressed in CD8^+^ T cells (108). The stimulations of the GITR signal as well as the CD137 signal enhanced T cells proliferation, raised the secretion of cytokines, and eliminated the suppressive effect of Treg cells (109–111). Compared to HCs, the expression of GITR was increased in patients with GPA and significantly correlated with disease activity (88). Giscombe et al. reported that, similar to animal tests, the expanded CD8^+^ T cells expressed more CD137 (89). The anti-GITR mAb exacerbated the disease severity in the murine model of collagen-induced arthritis (CIA) (112) and experimental autoimmune encephalomyelitis (EAE) (113). In contrast, the agonistic anti-CD137 mAb improved the CIA and EAE maybe by inducing expansion of CD11c^+^ CD8^+^ T cells (114, 115). Among the members of TNFRSF, CD137 was superior in increasing the secretion of cytokines by CD8^+^ T cells (116, 117). So far, the effects of inhibiting or stimulating GITR and anti-CD137 in the treatment of AAV are unknown. There is no doubt that this is a tempting field worthy of further exploration for AAV treatment.

## 4 Co-inhibitory signal pathways

**Figure 3:**
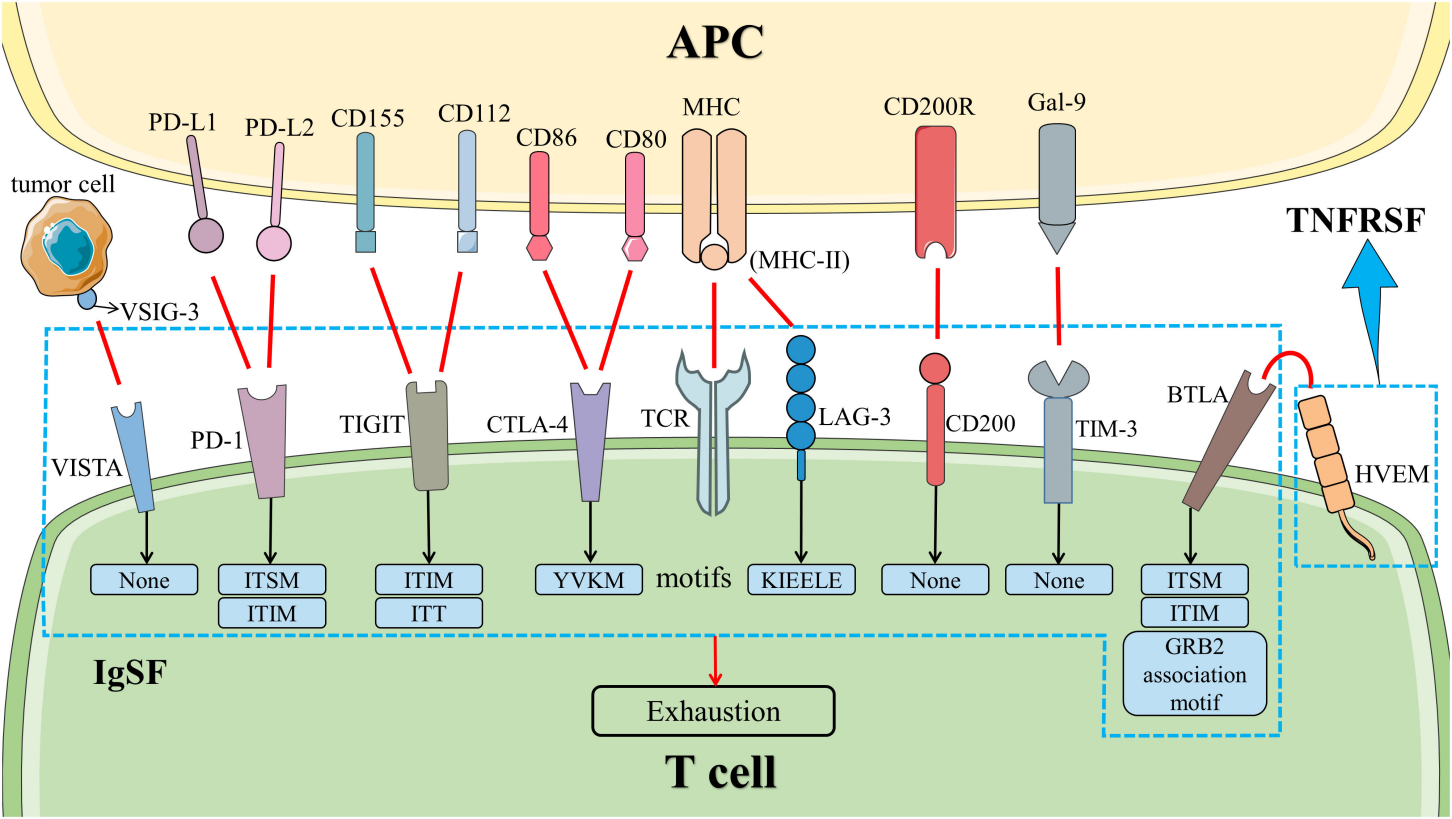
Co-inhibitory signal pathways in T cells. The co-inhibitory signal pathways induced the exhaustion of T cells. HVEM is a member of TNFRSF. Other molecules belong to IgSF. Except for LAG-3, VISTA, TIM-3, and CD200, all of them have inhibitory signal motifs in the cytoplasmic tail. LAG-3 has a unique KIEELE motif whose role is unclear, while VISTA, TIM-3, and CD200 do not have any motifs in the cytoplasmic tail. Abbreviation: APC, antigen-presenting cell; BTLA, B and T lymphocyte attenuator; CTLA-4, cytotoxic T lymphocyte-associated molecule 4; Gal-9, galactin-9; HVEM, herpesvirus entry mediator; IgSF, immunoglobulin superfamily; LAG-3, lymphocyte activation gene 3; MHC, major histocompatibility complex; PD-1, programmed cell death 1; PD-L1, programmed death ligand 1; TCR, T cell receptor; TIGIT, T‐cell immunoglobulin and ITIM domain; TIM-3, T cell immunoglobulin and mucin domain-containing protein 3; TNFRSF, tumor necrosis factor receptor superfamily; VISTA, V-domain immunoglobulin suppressor of T cell activation; VSIG-3, V-set and immunoglobulin domain containing 3. .

The exhaustion of T cells in AAV and other autoimmune diseases (AIDs) predicts advantageous clinical outcomes. The expressions of co-inhibitory molecules restrain the differentiation of non-exhausted T cells, indicating the importance of co-inhibitory molecules in the exhaustion of T cells (20). At present, the research on PD-1 and CTLA-4 signal pathways is reported widely, whose blockers have been used in the treatment of many malignant tumors. In the previous section, we summarized the association between AAV and inhibitors of PD-1, PD-L1, and CTLA-4. In this section, we described more details on the mechanisms and roles of PD-1 and CTLA-4 signal pathways in AAV. We assessed the therapeutic potential of other co-inhibitory molecules associated with the exhaustion of T cells as well (**Figure 3**).

### 4.1 PD-1 signal pathway

PD-1 is a co-inhibitory receptor expressed in many types of activated or exhausted immune cells (118). PD-1, a transmembrane protein with 288 amino acids, consists of an extracellular domain, a transmembrane domain, and an intracellular cytoplasmic tail. Although PD-1 belongs to the CD28 family, it has unique molecular characteristics. PD-1 has an IgV-like domain in the extracellular domain whereas an immunoreceptor tyrosine-based inhibition motif (ITIM) and an immunoreceptor tyrosine-based switch motif (ITSM) in the cytoplasmic tail (119). ITIM and ITSM mediated the inhibition signal of PD-1, while ITSM might be more important. It has been reported that only the mutation of ITSM affected the transduction of the PD-1 signal (120–122). With the bindings of PD-1 and its ligand, ITSM was phosphorylated, the SH2-containing protein tyrosine phosphatase 2 (SHP-2) was recruited, and the PI3K/AKT signal pathway was inhibited, which suppressed the activation of T cells and the production of pro-inflammatory cytokines (119, 123). RAS/MEK/ERK signal pathway, which is responsible for activating T cells, is another target of the PD-1 signal to suppress the activity of T cells and concurrent inflammation (119).

PD-L1 and PD-L2 are the ligands of PD-1. Both of them are members of B7 family, which are known as B7-H1 and B7-DC, respectively. The expression of PD-L1 is detected on many types of cells (T and B cells, DCs, VECs, placenta, eyes, and others) whereas PD-L2 is mainly limited to express on the surface of macrophages, DCs, and mast cells (119, 124). As PD-L1 and PD-L2 are close in the distance on the same chromosome, they are regulated similarly by inflammatory factors (IFN-1, IFN-2, TNF-α and ILs). In addition, PD-L1 is regulated with post-translational regulation and microRNAs, including but not limited to miR-513, miR-155, miR-34a, miR-142-5p, and miR-93 (118, 125, 126). PD-1 is not the only receptor for PD-L1 and PD-L2. PD-L1 can also bind to CD80 to inhibit the immune response of T cells (127), while PD-L2 may combine with repulsive guidance molecule B (RGMB) to impair respiratory tolerance (128). Therefore, targeting PD-L1 or PD-L2 is also important immunotherapy, and actually, PD-L1 inhibitors have been developed to treat malignant tumors.

Zhang et al. demonstrated that, in contrast to patients with GCA, PD-1^+^ T cells were not enriched at renal lesions in patients with GPA, suggesting that PD-1 might play a different role in different diseases (129). Similarly, Zeisbrich et al. measured the expression of PD-L1 in monocytes and found that the frequency of PD-L1^+^ monocytes was not related to renal involvement although these monocytes tended to decrease in active patients with AAV (130). A previous study revealed that the expression of PD-1 on Th cells was lower in patients with localized GPA than that in patients with systemic GPA (131). We then detected the expression of PD-1 in Tfh cells. Compared to HCs, the expression intensity of PD-1 in Tfh cells was higher in patients with MPO-AAV. We also found that the expression of ICOS/PD-1 instead of PD-1 was associated with the levels of MPO-ANCA (19), indicating that co-stimulatory and co-inhibitory molecules were involved in the activation of T cells together. Although the fusion proteins containing an anti-PD-1 single-chain variable fragment could improve symptoms of type 1 diabetes (TID) and EAE models (132), its effects in AAV have yet to be explored. Soluble ICs in PD-1 signaling pathway also play an important role in AAV. It was observed that although the serum concentration of soluble PD-L2 in active AAV was lower than that in HCs, its level was significantly increased after treatment (57). In addition, the levels of serum soluble PD-1 (sPD-1) was higher in MPO-AAV (133). Noting that sPD-1 could restrain the exhaustion of T cells by binding to PD-L1 in cell membranes (134), sPD-1 is therefore likely an effective therapeutic target in MPO-AAV. Significantly, Targeting the PD-1 signal pathway alone may be less effective because of the poorer inhibitory impact on PD-1 of T cells (131). This may explain why PD-1 inhibitors induce fewer irAEs than CTLA-4 inhibitors. Compared to PD-1, PD-L1 may be a better target. The frequency of PD-L1^+^ monocytes is negatively correlated with the level of ANCA (130), so increasing the expression of PD-L1 may reduce the level of ANCA and improve disease activity. In lupus nephritis mice models, the recombinant adenovirus containing the full-length PD-L1 gene improved the renal lesions (135). In the same experiment, the anti-ICOSL mAb was also added to reinforce this process (135). As the results we found, targeting both ICOS and PD-1 in patients with AAV may be more effective.

### 4.2 CTLA-4 signal pathway

CTLA-4 is transiently expressed in the activated T cells. Beforehand, it localized in intracellular compartments of naïve T cells (136, 137). The gene expressing CTLA-4 is located in the same chromosomal region (2q33-34) as the gene expressing CD28, so CTLA-4 and CD28 exhibit a significant homology (138). CTLA-4 and CD28 bind to the same ligand but transduce an opposite signal. CTLA-4 leads to co-inhibitory signaling whereas CD28 provides the co-stimulatory signal. As a member of IgSF, CTLA-4 is also a transmembrane protein that contains an IgV-like domain in the extracellular domain and a YVKM motif in the intracellular cytoplasmic tail (139, 140). The IgV-like domain is the site that binding to CD80 and CD86 for CTLA-4, and the YVKM motif is important for CTLA-4 signal transduction in T cells. The interaction of the phosphorylated YVKM motif with SHP2 and serine/threonine protein phosphatase 2A (PP2A) dephosphorylated the TCR-CD3ζ complex so that the activated signal from TCR was suppressive (21). On the other hand, the phosphorylation of the YVKM motif inhibited the interaction with the clathrin-associated adaptor complex AP-2, resulting in the internalization of CTLA-4 (141). CTLA-4 expressed on the surface of activated T cells captured and degraded CD80 and CD86 by trans-endocytosis to inhibit the co-stimulatory signal from CD28 (142). Ultimately, CTLA-4 suppressed the activation of T cells.

In patients with GPA, the expression of CTLA-4 in T cells was found to be elevated, and to be related to disease severity. After stimulating with phytohaemagglutinin (PHA), the expression of CTLA-4 in T cells in patients with GPA did not increase, suggesting that the activation of T cells in patients with GPA was persistent (143). As mentioned above, Abatacept bind to CD80 and CD86 to inhibit the activation of T cells, and its efficacy in patients with GPA has been reported (59). Although agonistic CTLA-4 antibodies have not yet been successfully developed, directly targeting CTLA-4 may be less effective because of the CTLA-4 endocytosis. It is probably more effective to inhibit the bindings of CD28 to CD80 and CD86. In addition, polymorphisms of the CTLA-4 gene were associated with GPA (144–146), while they were not related to MPA in Japanese patients (147), indicating that there were race differences in CTLA-4 polymorphism. A meta-analysis showed that CTLA-4 (AT)_86_ and CTLA-4 (AT)_106_ were significantly associated with AAV in the Caucasian patients instead of the Asian patients (144). Therefore, when targeting CTLA-4, genetic variation should be considered to avoid invalid treatment.

### 4.3 Other co-inhibitory signal pathways

T cell Ig and mucin domain-containing protein 3 (TIM-3), as a co-inhibitory receptor for Th1, was reported in 2002 to be associated with the severity of EAE (148). After that, it was shown that TIM-3 was expressed in Treg cells, DCs, natural killer (NK) cells, and macrophages (149, 150). Similar to other members of IgSF, TIM-3 contains an IgV domain, a mucin domain, a transmembrane domain, and a cytoplasmic tail lacking inhibitory signaling motifs (151). TIM-3 binds to ligands through the IgV domain, and five conserved tyrosine residues at the cytoplasmic tails trigger the signaling downstream (152). Galactin-9 (Gal-9), phosphatidyl serine (PtdSer), high mobility group protein B1 (HMGB1), and carcinoembryonic antigen cell adhesion molecule 1 (CEACAM-1) are known ligands of TIM-3. Gal-9 is the earliest discovered as well as the most explored ligand. TIM-3-Gal-9 signal pathway induced intracellular calcium flux, apoptosis, and the suppression of Th1 (153). PtdSer and HMGB1 did not directly suppress the activation of T cells, but rather affected the immune responses of DCs (154, 155). CEACAM-1 promoted the exhaustion of T cells through cis- and trans-interactions with TIM-3 (156). In patients with AAV, the expression of TIM-3 was significantly reduced on DCs, and blocking TIM-3 enhanced the expression of DC cytokines. Also, there were no differences in the expression of TIM-3 on the surface of different T cell subtypes (CD4^+^ T cells and CD8^+^ T cells) between MPO-AAV and HCs (157). Further explorations might be needed to assess the expression of TIM-3 on Th and Treg cells. Studies have shown that the serum concentrations of soluble TIM-3 correlated with the diseases state of AAV (57, 133), i.e. it was increased in active AAV (57), and could predict the relapse in PR3-AAV with rituximab treatment (133). However, the specific mechanism of soluble TIM-3 in AAV remains to be further examined before considering as a therapeutic target. Yoon et al. demonstrated that the serum Gal-9 levels were independently related to disease activity in patients with AAV (158). In mice models of CIA and SLE, injection of Gal-9 improved symptoms (159, 160). Although targeting the TIM-3 signal pathway has a potential efficacy in AAV, the problem is the need to clarify in which immune cells the TIM-3 signal pathway is more dominant to confirm the effectiveness. Another problem is that TIM-3 is not the only receptor for its ligand, so targeting the ligands of TIM-3 may not completely enhance TIM-3 signaling.

B and T lymphocyte attenuator (BTLA, also known as CD272) is mainly expressed in B and T cells, especially in naïve B cells and Th1 (161). BTLA is a co-inhibitory molecule with similar structures to PD-1 and CTLA-4 (162). Similar to PD-1, ITSM and ITIM in the cytoplasmic tail of BTLA inhibit the activation of T cells. However, unlike PD-1, SHP-1 rather than SHP-2 is mainly recruited by BTLA (163, 164). The third signal motif in the cytoplasmic tail of BTLA is the GRB2 association motif, which binds to GRB2 and p85 subunits of PI3K and induces the activation of T cells (165). Accordingly, BTLA transmits bidirectional signaling. Herpesvirus entry mediator (HVEM, also known as TNFRSF14) is a ligand of BTLA. HVEM is a member of TNFRSF, which is expressed in T cells, B cells, NK cells, monocytes, and neutrophils (166). There are two types of interaction between HVEM and BTLA. When BTLA and HVEM interacted in the same T lymphocyte, a cis complex was formed, inhibiting HVEM-dependent NF-κB activation (167). When BTLA or HVEM was expressed in APCs, trans interaction provided a co-stimulatory signal (168). Therefore, the regulation of the BTLA-HVEM signal pathway in AIDs is complex. In patients with remission AAV, the expression of BTLA was decreased only on double negative T-cells (CD3^+^CD4^-^CD8^-^). *In vitro* experiments, it has been shown that agonistic anti-BTLA antibody inhibit the activation and proliferation of T cells, especially Th17 (169), suggesting that targeting BTLA to inhibit the activation of T cells may be one of the future therapeutic directions of AAV.

V-domain Ig suppressor of T cell activation (VISTA), also known as PD-1 homolog (PD-1H), is a member of IgSF first discovered in 2011 (170). Different from other members of IgSF, VISTA has four additional invariant cysteines (170, 171). Subsequently, the same laboratory confirmed that VISTA in humans is a co-inhibitory molecule, which inhibits the proliferation of T cells and the production of cytokines (172). VISTA is mainly expressed in hematopoietic cells, especially in CD11b^hi^ myeloid cells. Within the T cell compartment, the expression of VISTA was highest in naïve T cells and FoxP3^+^ Treg cells (171, 172). It is certain that VISTA is a ligand for T cells (170, 172). Moreover, it may also be a receptor in T cells to transmit inhibitory signals (173). Wang et al. demonstrated that V-set and Ig domain containing 3 (VSIG-3) is a ligand of VISTA. The binding between VISTA and VSIG-3 induced the inhibitory effects (174). It is a pity that VSIG-3 is mainly expressed in tumor cells but not in normal immune cells (174). Currently, targeting VISTA is explored in AIDs. It was reported that VISTA KO mice developed SLE (175) and EAE (176). In mice models with SLE, agonistic VISTA mAb improved symptoms (175). In patients with AAV, VISTA was expressed in mononuclear phagocytes, CD4^+^ T cells, and CD8^+^ T cells. Compared to patients with a lower expression of VISTA, patients with a higher expression of VISTA might have a higher risk of renal progression (177). Therefore, targeting VISTA may inhibit disease severity in patients with AAV, which needs more experiments to confirm.

T‐cell Ig and ITIM domain (TIGIT), CD200, and lymphocyte activation gene 3 (LAG-3) are popular co-inhibitory molecules in recent studies. CD155 and CD112 are ligands of TIGIT, binding to CD226 as well (152). CD200 binds to CD200Rs, especially CD200R1 (178). Besides MHC-II, fibrinogen-like protein 1 (FGL-1) was also discovered to be a ligand for LAG-3 (179, 180). These signal pathways transmit inhibitory signals. As a result, their roles in AIDs are noted. TIGIT-Ig fusion protein has revealed the therapeutic effects in mice with SLE (181). In EAE mice models, agonistic TIGIT mAb as well as CD200-Fc fusion protein improved disease severity (182, 183). CD200-Fc fusion protein reduced the disease severity of CIA at the clinical and histologic levels (184). Remarkably, LAG-3-deficient mice do not induce AIDs. After exposure to mercury (Hg), it not only had increased susceptibility to AIDs but also did not respond to tolerance induction (185). In AAV, there are no relevant reports about the influences of TIGIT, CD200, and LAG-3, so it is unknown whether targeting these signal pathways will improve disease severity.

## 5 Conclusion

The pathogenesis of AAV involves multiple aspects of innate immunity and adaptive immunity in which the role of T cells is pivotal and complex. With the understanding of the IC molecules, its importance will be confirmed further in AAV. Firstly, some ICIs used for malignant tumors induced the attack or relapse of AAV. Secondly, inhibiting the co-stimulatory signal pathways or enhancing the co-inhibitory signal pathways inhibited the activation and proliferation of T cells so that AIDs could be improved. In AAV, although it is demonstrated that Abatacept is effective in clinical trials, the efficacy of targeting other ICs has not been demonstrated. Thirdly, since co-stimulatory and co-inhibitory molecules work together to regulate T cells, it may be more reasonable to target multiple ICs simultaneously in severe or refractory cases. The questions to be aware of are that targeting ICs may increase the risk of tumors and infections, and different ICs are dominant in different subsets of T cells, so it is required to precise dosing and localization. In conclusion, targeting ICs has therapeutic potential, and more preclinical research is needed to clarify their effectiveness and safety in AAV treatment.

## Author contributions

PSCL originated the topic and revised the manuscript. ZS wrote the outline, organize and supervise the writing. MP and HZ wrote the original text and figures draft. RJ searched literatures and made the table. All authors contributed to the article and approved the submitted version.

## Glossary

**Table:**

AAV	ANCA associated vasculitis
AIDs	autoimmune diseases
ANCA	anti-neutrophil cytoplasmic antibody
APC	antigen-presenting cells
BCR	B cell receptor
BTLA	B and T lymphocyte attenuator
C5a	fragment a of fifth complement
cAP	complement alternative pathway
CEACAM-1	carcinoembryonic antigen cell adhesion molecule 1
CIA	collagen-induced arthritis
CTL	cytotoxic T lymphocytes
CTLA-4	cytotoxic T lymphocyte-associated molecule 4
DCs	dendritic cells
EAE	experimental autoimmune encephalomyelitis
EGPA	eosinophilic granulomatosis with polyangiitis
Gal-9	Galactin-9
GCA	giant cell arteritis
GCs	germinal centers
GITR	glucocorticoid induced TNF receptor
GPA	granulomatosis with polyangiitis
GRB2	growth factor receptor-bound protein 2
GVHD	graft-versus-host disease
HCs	healthy controls
HMGB1	high mobility group protein B1
HSP	Henoch-Schönlein purpura
HVEM	herpesvirus entry mediator
IC	immune checkpoint
ICIs	immune checkpoint inhibitors
ICOS	inducible T-cell co-stimulator
ICOSL	inducible T-cell co-stimulator (ICOS) ligand
IFN	interferon
Ig	immunoglobulin
IgSF	immunoglobulin superfamily
IgV	Ig variable
IL	interleukin
irAEs	immune-related adverse events
LAG-3	lymphocyte activation gene 3
LCK	lymphocyte cell-specific protein-tyrosine kinase
mAb	monoclonal antibody
MHC	major histocompatibility complex
MNPs	mononuclear phagocytes
MPA	microscopic polyangiitis
MPO	myeloperoxidase
mTOR	mammalian target of rapamycin
NETs	neutrophil extracellular traps
NFAT	nuclear factor of activated T cells
NF-κB	nuclear factor-κB
NHP	non-human primate
NIK	NF-κB–inducing kinase
NIKR	tumor necrosis factor receptor
NK	natural killer cell
OX40L	OX40 ligand
PD-1	programmed cell death 1
PD-L	programmed death ligand
PI3K	phosphatidylinositol 3-kinase
PR3	proteinase-3
PtdSer	phosphatidyl serine
Rh-irAEs	rheumatic immune-related adverse events
sCD28	soluble CD28
SH2	Src homology-2
SHP-2	SH2-containing protein tyrosine phosphatase 2
SLE	systemic lupus erythematosus
SLEDAI	SLE disease activity index
sPD-1	soluble PD-1
TAK	Takayasu’s arteritis
TCR	T cell receptor
Teff	effector T
Tfh	follicular helper T cells
Th	helper T cells
THD	TNF homology domain
TIGIT	T‐cell Ig and ITIM domain
TIM-3	T cell Ig and mucin domain-containing protein 3
TNF	tumor necrosis factor
TNFR	tumor necrosis factor receptor
TNFRSF	tumor necrosis factor receptor superfamily
TRAF	tumor necrosis factor receptor-associated factor
Treg	regulatory T cells
VECs	vascular endothelial cells
VISTA	V-domain Ig suppressor of T cell activation
VSIG-3	V-set and Ig domain containing 3
